# Meta-analysis of acupuncture for relieving non-organic dyspeptic symptoms suggestive of diabetic gastroparesis

**DOI:** 10.1186/1472-6882-13-311

**Published:** 2013-11-09

**Authors:** Mingxing Yang, Xiumin Li, Suhuan Liu, Zhipeng Li, Mei Xue, Dehong Gao, Xuejun Li, Shuyu Yang

**Affiliations:** 1 Xiamen Diabetes Institute, Xiamen, China; 2 Department of Endocrinology and Diabetes, the First Affiliated Hospital of Xiamen University, Xiamen, China; 3 Department of Electronic Science, School of Physics and Mechanical & Electrical Engineering, Xiamen University, Xiamen, China

**Keywords:** Acupuncture, Dyspeptic symptoms, Diabetic gastroparesis, Gastroprokinetic agent, Meta-analysis

## Abstract

**Background:**

Acupuncture is widely used to treat diabetic patients with dyspeptic symptoms suggestive of gastroparesis in China. We conducted this systematic review of randomized controlled trials (RCTs) to evaluate the efficacy of acupuncture for diabetic gastroparesis (DGP).

**Methods:**

We searched PubMed, EMBASE, Cochrane Central Register of Controlled Trials (CENTRAL) and four Chinese databases including China National Knowledge Infrastructure (CNKI), VIP Database for Chinese Technical Periodicals, Chinese Biomedical Literature Database (CBM) and WanFang Data up to January 2013 without language restriction. Eligible RCTs were designed to examine the efficacy of acupuncture in improving dyspeptic symptoms and gastric emptying in DGP. Risk of bias, study design and outcomes were extracted from trials. Relative risk (RR) was calculated for dichotomous data. Mean difference (MD) and standardized mean difference (SMD) were selected for continuous data to pool the overall effect.

**Results:**

We searched 744 studies, among which 14 RCTs were considered eligible. Overall, acupuncture treatment had a higher response rate than controls (RR, 1.20 [95% confidence interval (CI), 1.12 to 1.29], *P* < 0.00001), and significantly improved dyspeptic symptoms compared with the control group. There was no difference in solid gastric emptying between acupuncture and control. Acupuncture improved single dyspeptic symptom such as nausea and vomiting, loss of appetite and stomach fullness. However, most studies were in unclear and high risk of bias and with small sample size (median = 62). The majority of the RCTs reported positive effect of acupuncture in improving dyspeptic symptoms.

**Conclusions:**

The results suggested that acupuncture might be effective to improve dyspeptic symptoms in DGP, while a definite conclusion about whether acupuncture was effective for DGP could not be drawn due to the low quality of trials and possibility of publication bias. Further large-scale, high-quality randomized clinical trials are needed to validate this claim and translate this result to clinical practice.

## Background

Diabetic gastroparesis (DGP) is a common autonomic neuropathy which affects more than 5% diabetic patients [1,2]. It not only affects nutritional state but also adversely impacts on glycemic control and quality of life in diabetes. Because of the increasing prevalence of diabetes, diabetic gastroparesis is expected to increase in the next 20 years, especially in China which has more than 200 million patients with diabetes and prediabetes [3].

Gastroprokinetic agents such as domperidone, cisapride and mosapride are widely used to treat diabetic gastroparesis all over world including China, since the delayed gastric emptying is considered as a potential contributor to this functional dyspepsia. In china, acupuncture and traditional Chinese medications have been used to treat gastrointestinal tract disorders over 1500 years. It is documented in ancient Chinese medical books that acupuncture can treat many digestive symptoms such as stomach fullness, nausea, abdominal pain, vomiting, loss of appetite and bloating. Although Chinese ancient doctors were not able to give a definite diagnosis of disease, it can be deduced that these digestive symptoms might be involved in diabetic gastroparesis. In recent years, with the accumulating evidence of acupuncture for gastrointestinal tract and definite diagnosis of diabetic gastroparesis, some acupuncturists in China have realized that acupuncture might have potential effects in treating diabetic gastroparesis. It has been recently shown that acupuncture improved gastric motility in experimental animals [4-7], and improved gastrointestinal motility and promoted gastric emptying in human [8,9]. During the last 20 years, Chinese acupuncturists performed many clinical studies to evaluate the effectiveness of acupuncture for diabetic gastroparesis. However, most of these studies had a small sample sizes, a conclusion of pooled effect about acupuncture on DGP remained to draw. The present study was therefore conducted to assess the quality of trials and the effect of acupuncture on treating diabetic gastroparesis.

## Methods

### Literature search

We presented this report in accordance with the principals of Preferred Reporting Items for Systematic Reviews and Meta-Analyses [10]. Literature search was performed using three English databases including PubMed, EMBASE and CENTRAL, and four Chinese databases, including China National Knowledge Infrastructure (CNKI), VIP Database for Chinese Technical Periodicals, Chinese Biomedical Literature Database (CBM) and WanFang Data from their inception to January 2013. Searching terms for PubMed were as follows: ((diabet* AND “gastric emptying”) OR (gastroparesis AND diabet*)) AND (acupuncture OR electroacupuncture OR acup* OR “acupuncture therapy” OR “scalp acupuncture” OR “eye acupuncture” OR “abdomen acupuncture” OR “ear acupuncture”). These search terms were slightly adjusted for other databases. No language restriction was applied.

### Study selection

Studies meeting the following criteria were included: the study was a randomized controlled clinical trial; the intervention of interest was acupuncture-related methods such as acupuncture, electroacupuncture (EA), scalp acupuncture, eye acupuncture, ear acupuncture or abdomen acupuncture; the control group was treated with sham acupuncture or gastroprokinetic agents; all participants, regardless of age, gender and ethnicity, were diagnosed as diabetes with dyspeptic symptoms excluding gastric outlet obstruction or ulceration by upper endoscopy, ultrasound or barium X-ray. The primary outcome measurement was gastroparesis Cardinal Symptom Index (GCSI) [11] or a similar scale [12] to score dyspeptic symptoms, and the secondary outcome measure was gastric emptying detected by scintigraphy or radio-opaque markers [13]. Studies, in which the main intervention was moxibustion or acupuncture combined with Chinese materia medica, were excluded because the reported effects in these studies did not arise from needle-penetrating acupuncture or the effects were confounded by Chinese materia medica, while trials in which the main intervention was acupuncture combined with acupuncture-related assistant techniques were included because the effect of these studies was from stimulation of acupoints by acupuncture or its assistant techniques. Studies defined effect index “significant improvement” as loss of all dyspeptic symptoms and “improvement” as decrease of dyspeptic symptoms but not based on the change of total scores of dyspeptic symptoms were excluded since these outcome measures might be subjective.

### Data extraction and assessment of the risk of bias

Two authors (MX Yang and XM Li) independently reviewed the titles and abstracts to assess the eligibility of the references according to the criteria mentioned above. A standardized data extraction process was used to collect the following information: title, authors, year of publication, characters of population, number of participants, location, and duration of interventions, outcomes, side effects, follow-up and risk of bias. We also described the type of acupuncture and the control-intervention. Risk of bias in trials were evaluated according to the Cochrane collaboration’s update tool for assessing the risk of bias, published in the Cochrane handbook for systematic reviews of interventions (Version 5.1.0, updated March 2011) [14]. This tool can be used to systematically assess the risk of bias of clinical trials about selection bias, performance bias, detection bias, attrition bias, reporting bias and other bias with three grades: high, unclear and low risk of bias. In data extraction, any disagreement was resolved by discussion. When the methods in some studies were not described clearly such as the generation of random sequence, we tried to contact the first or corresponding author to get additional information by letters or e-mails.

### Statistical analysis

Statistical analysis was carried out using Review Manage software (V5.1.4, Nordic Cochrane Center, Copenhagen, Denmark). Dichotomous data was presented as relative risk (RR) with statistical method Mantel-Haenszel (M-H) and continuous data as mean difference (MD) and standardized mean difference (SMD) with inverse variance (IV) method, both with 95% confidence interval (CI). Heterogeneity across studies was determined by chi-squared (χ^2^) test (significance level at *P* < 0.10). In addition, the *I*^2^ statistic, a quantitative measure of heterogeneity among studies [15], was also calculated and the significant level was set *I*^2^ < 50%. Random effect model was used to calculate the pooled effect when there was significant heterogeneity among trials. If *I*^2^ > 75%, qualitative description was provided. Otherwise, the fixed effect model was selected to pool the data. We used SMD to combine the effect of acupuncture on dyspeptic symptoms since some trials measured the severity of symptoms by GCSI [11] and others used a similar scale [12]. For dichotomous data, cases that dropped out or with missing data were included by counting them as treatment failure in the acupuncture group and success in control group (worst-case scenario analysis). Potential publication bias was examined by funnel plot, i.e., a graphical display of the standard error of the intervention effect estimate plotted against effect size [16].

GRADEpro 3.6 [17] was used to produce GRADE evidence profile to summarize the confidence in estimates of acupuncture effects for patients and the strength of recommendation.

Subgroup analysis was conducted in term of control type (e.g. domperidone, cisapride and mosapride) and symptoms (e.g. nausea, loss of appetite and stomach fullness). In addition, sensitivity analysis was also employed on those studies with low risk of selection bias as previous reported [18].

## Results

### Studies description

**Figure 1:**
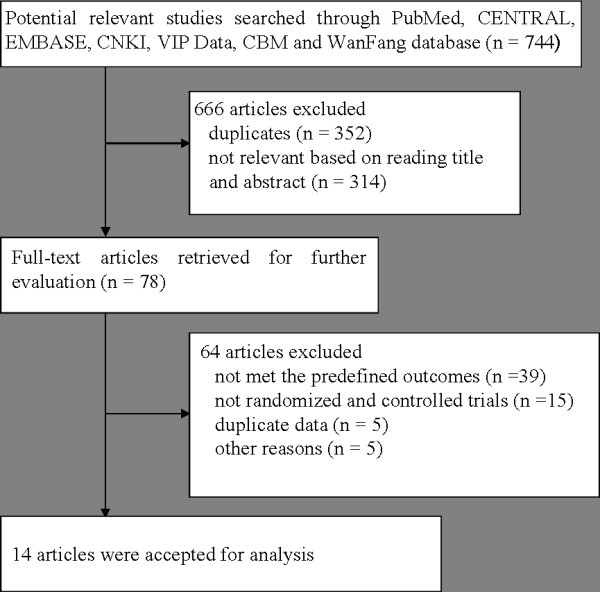
Flow diagram of study selection.

Our initial searches identified 744 relevant studies concerning acupuncture and acupuncture-related treatment for diabetes with dyspeptic symptoms. 666 articles were excluded because they were duplicates (n = 352) or not clinical studies (n = 314) based on reading the titles and abstracts (Figure 1). Full-text of remaining 78 articles published in Chinese or English were retrieved for further assessment. Of these, 64 articles were excluded because they did not meet our inclusion criteria or were not randomized and controlled trials, duplicates and other reasons. Finally, the remaining 14 RCTs [19-32] were considered eligible which reported randomly assignment of patients (n = 948) to acupuncture and control group (one sham-EA [24], six domperidone [20,21,23,28-30], two cisapride [22,25] and five mosapride [19,26,27,31,32]).

All studies were conducted from 2001 to 2011. The median sample size of these trials was 62 patients, varying from 19 [24] to 120 [21]. Of 14 trials, six studies used acupuncture [20,22,25,26,28,29], five studies used EA [19,21,24,31,32], one study used warm acupuncture [30], one trial used acupuncture combined with acupoint application [23] and another study used acupuncture combined with chiropractic [27]. Studies in which the interventions were acupuncture combined with acupoint application and chiropractic were included because acupoint application and chiropractic were considered as assistant techniques to acupuncture and the reported effects of these studies were mainly from needle-penetrating acupuncture on acupoints. Details of studies were tabulated in Table 1.

**Table 1:** The characteristics of the included trials

**Study ID**	**n (T/C)**	**Age (T/C, years)**	**Acupuncture intervention**	**Acupoints**	**Control intervention**^**c**^	**Outcomes**	**Drop-out**	**Follow up**
Wang 2008 [24]	19 (9/10)	57.7 ± 7.4/57.1 ± 9.9	EA, 2 sessions per week, 2 weeks	ST36, LI4	Sham-EA, 2 sessions per week, 2 weeks	Dyspeptic symptoms, gastric emptying	Yes	Two weeks
Ge 2010 [20]	60 (30/30)	40-61/42-60	Acupuncture, 5 sessions per week, 4 weeks	CV12, ST36, PC6	Domperidone, 10 mg, bid, 4 weeks	Response rate	No discription	No discription
Shen 2010 [23]	60 (30/30)	52 ± 10.5/50 ± 10.2	Acupuncture and acupoint application^d^, 6 sessions per week, 4 week	PC6, CV12, CV6, ST36, SP6	Domperidone, 10 mg, tid, 4 weeks	Response rate	No discription	No discription
Wang 2010 [27]	70 (35/35)	37-84/40-85	Chiropractics and acupuncture, 1 sessions per day, one month	BL20, BL21, BL18, BL23, PC6, ST36, SP6, CV12	Mosapride, 5 mg, tid, one month	Dyspeptic symptoms, response rate	No discription	No discription
Zeng 2008 [28]	60 (30/30)	52 ± 12/51 ± 15	Acupuncture, one sessions per day, 4 weeks	CV12, ST36, PC6, SP6	Domperidone, 10 mg, tid, 4 weeks	Dyspeptic symptoms, response rate	Yes^b^	No discription
Zhang 2007 [29]	72 (36/36)	47.26 ± 5.13/48.31 ± 6.57	Acupuncture, two sessions per day, thirty days	BL21, CV12, BL20, LR13, BL23, BL18, LR14, GB25, ST25, ST36	Domperidone, 10 mg, tid, thirty-four days	Dyspeptic symptoms, response rate	No discription	No discription
Zheng 2010 [30]	80 (40/40)	44.7 ± 8.9/43.9 ± 9.1	Warm acupuncture, 5 sessions per week, 4 weeks	CV12, ST36, PC6	Domperidone, 10 mg, tid, 4 weeks	Response rate, gastric emptying	No discription	No discription
Han 2001 [21]	120 (60/60)	52.12 ± 2.61/51.65 ± 2.53	EA, 1 sessions per day, 2 weeks;	ST36, ST25, PC6, ST39, CV12	Domperidone, 10 mg, tid, 2 weeks	Dyspeptic symptoms, response rate	No discription	No discription
Li 2006 [22]	60 (30/30)	40-69/42-70	Acupuncture, one sessions per day, fifteen days	ST36, CV12, ST25, BL21, BL20, LR3, BL23, PC6	Cisapride, 10 mg, tid, fifteen days	Dyspeptic symptoms, response rate	Yes^b^	No discription
Wang 2007 [26]	63 (31/32)	57.67 ± 6.55 58.03 ± 7.99	Acupuncture, one sessions per day, thirty days	BL21, CV12, BL20, LR13, BL23, BL18, LR14, GB25, ST25, ST36	Mosapride, 5 mg, tid, thirty days	Dyspeptic symptoms, response rate, gastric emptying	Yes^b^	No discription
Wang 2009^a^[25]	70 (35/35)	26-65/28-69	Acupuncture, one sessions per day, thirty days	CV17, CV13, CV12, CV4, CV10, CV8, CV 6	Cisapride, 10 mg, tid, thirty days	Gastric emptying	No discription	No discription
Chen 2008 [19]	60 (30/30)	57.67 ± 2.04/59.77 ± 2.21	EA, 5 sessions per week, three weeks	CV12, ST36, ST25, ST21, ST37	Mosapride, 5 mg, tid, three weeks	Dyspeptic symptoms, response rate, gastric emptying	Yes^b^	No discription
Zhao 2011 [31]	60 (30/30)	54.77 ± 12.26/54.80 ± 9.42	EA, 5 sessions per week, two weeks	ST36, CV12, ST25, ST21, ST37	Mosapride, 5 mg, tid, two weeks	Dyspeptic symptoms, response rate, gastric emptying	Yes^b^	No discription
Chen 2005 [32]	60 (30/30)	58.83 ± 11.80/61.13 ± 9.01	EA, 5 sessions per week, two weeks	CV12, ST21, ST25, BL21, ST36	Mosapride, 5 mg, tid, two weeks	Dyspeptic symptoms, response rate, gastric emptying	Yes^b^	No discription

Key: ^a^, three-arm trial; ^b^, patients with incomplete data or bad compliance were exclude in these studies; ^c^, drugs were took orally by patient; ^d^, acupoint application is a traditional therapy which is based on the Chinese meridian theory, sticking some excitive Chinese medicine such as *Herba Asari* to acupoint to stimulate local skin of acupoint to regulate body blood and energy with the objective to prevent and treat disease.

T/C, treatment/control; EA, electroacupuncture; bid, bis in die; tid, ter in die.

Of 14 RCTs, only one trial [24] reported a follow-up of two weeks, but the follow-up data was not provided. One study [26] reported three lost cases and we analyzed this data by a worst case model.

### Risk of bias

**Figure 2:**
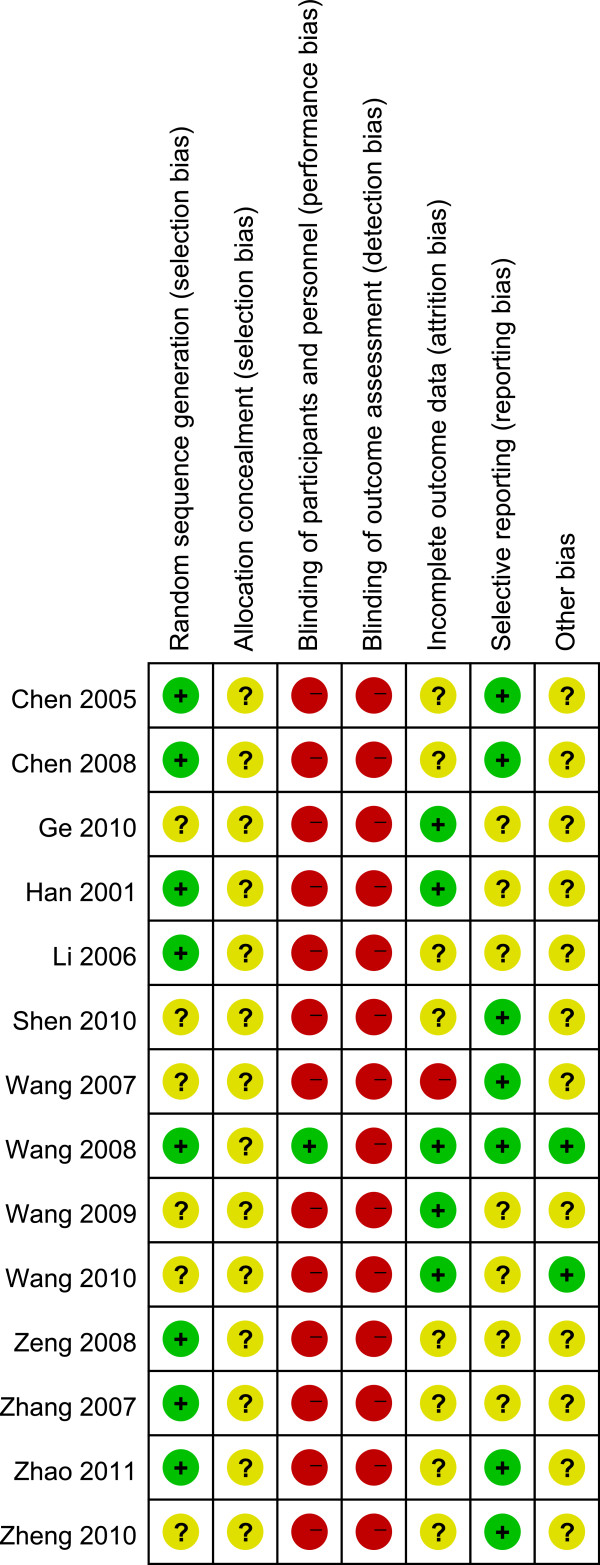
**Risk of bias summary.** Key: red circle symbolizes high risk of bias, green circle symbolizes low risk of bias, yellow circle symbolizes unclear risk of bias.

Assessment of the risk of bias was based on the original descriptions of random sequence generation, allocation concealment, blinding, incomplete outcome data and other bias. Figure 2 showed the summary of risk of bias of the included trials for acupuncture response rate and symptom improvement. In general, random sequences in 8 studies [19,21,22,24,28,29,31,32] out of 14 trials were generated correctly with clear descriptions (low risk of bias), but no trial gave a description of concealed allocation in their studies (unclear risk of bias). One trial [24] reported blinding of participants with sham-EA. No trial provided a description of blinding outcome assessors. Considering effect index and scores of symptoms were based on patient-reported outcome, all studies were assessed as high risk of bias in blinding of outcome assessment (Figure 2). Five studies [20,21,24,25,27] were assessed as low risk of bias associated with incomplete outcome data. Only one studies [26] reported drop-out, but intention to treat analysis (ITT) was not used in their data analysis. Six trials [19,22,28-31] excluded the cases who had incomplete data, bad compliance or drop-out. All trials reported baseline comparison of age, sex and duration of diabetes, but only seven studies [19,24,26-29,31] gave baseline comparison of severity of gastric dyspeptic symptoms which were assigned as low risk of bias.

We contacted the first or corresponding authors of primary study to get details about study methods such as random sequence generation and blinding. Unfortunately, we received only two responses.

### Response rate to acupuncture

Eight RCTs [20-22,26-30] involving 585 diabetic patients were identified to observe the response rate of acupuncture in improving dyspeptic symptoms. Scales were used in all studies to determine the score change of dyspeptic symptoms such as bloating, stomach fullness, nausea, vomiting, retching, and loss of appetite after acupuncture. Seven trials [21,22,26-30] classified the effect of acupuncture on dyspeptic symptoms into three levels, “significant improvement”, “improvement”, “no improvement” according to the effect index calculated by (total scores before treatment – total scores after treatment)/total scores before treatment × 100%. Significant improvement, improvement, no improvement were defined as effect index >75%, >25%, and <25%, respectively. One trial [20] reported the effect index by four level, “clinical cure” (effect index ≥95%), “significant improvement”, “improvement”, “no improvement”. For overall analysis, we transformed these outcomes into dichotomous data by grouping together “significant improvement”, “improvement” and “clinical cure” as “effective” and defined “no improvement” as “ineffective”.

**Figure 3:**
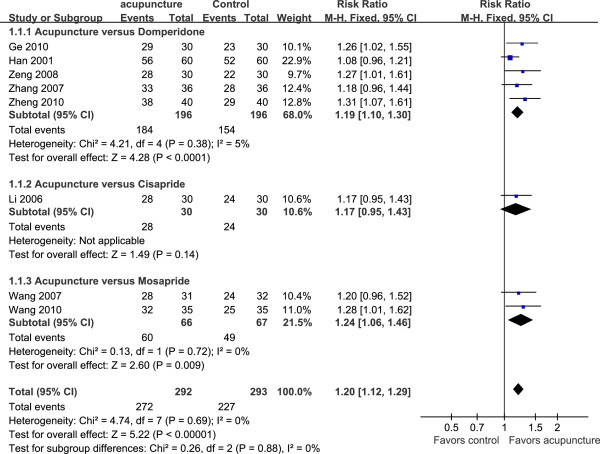
**Efficacy of response rate to acupuncture compared with gastroprokinetic agents in 8 studies.** CI, confidence interval; df, degree of freedom; M-H, Mantel-Haenszel test.

Pooled effect from five trials [20,21,28-30] involving 392 patients implied that acupuncture was more effective at response rate than domperidone (RR, 1.19 [95% CI, 1.10 to 1.30], *P* < 0.0001) with no heterogeneity (*I*^2^ = 5%, df = 4, χ^2^ = 4.21, *P* = 0.38) (Figure 3). Among these 5 trials, the study with the largest sample size had 120 patients [21]. One trial [21] applied EA, another trial [30] used warm acupuncture. The number of session was 14 [21,28] to 60 [29].

One trial [22] involving 60 patients suggested that there was no difference between acupuncture and cisapride treatment (RR, 1.17 [95% CI, 0.95 to 1.43], *P* = 0.14).

There were two trials [26,27] evaluated acupuncture in 133 patients controlled by mosapride. One trial [26] reported three lost patients, one from acupuncture group and two from control group. We counted one case as treatment failure in acupuncture group and two cases as treatment success in control group. The combined result of these two trials showed that acupuncture had a higher response rate than mosapride (RR, 1.24 [95% CI, 1.06 to 1.46], *P* = 0.009) (Figure 3). No significant heterogeneity was found between these two trials (*I*^2^ = 0%, df = 1, χ^2^ =0.13, *P* = 0.72).

The pooled effect of 8 RCTs showed that acupuncture was more effective than gastroprokinetic agents in response rate (RR, 1.20 [95% CI, 1.12 to 1.29], *P* < 0.00001, n = 585) (Figure 3) with no significant heterogeneity across studies (*I*^2^ = 0%, df = 7, χ^2^ = 4.74, *P* = 0.69).

We could not use funnel plot to detect publication bias and small study effect because of the small number of included studies.

### Sensitivity analysis of response rate to acupuncture

Sensitivity analysis was performed by excluding studies with high risk of bias of random sequence generation, because high risk of selection bias might overestimate acupuncture effect. This analysis was limited to four trials [21,22,28,29] and the results showed that the RRs of acupuncture against domperidone and cisapride were 1.15, ([95% CI, 1.04 to 1.26], *P* =0.006, three trials [21,28,29], fixed model) and 1.17 ([95% CI, 0.95 to 1.43], *P* =0.14, one trial [22]), respectively. Overall effect of acupuncture vs domperidone and cisapride was still favorable (RR =1.15, [95% CI, 1.05 to 1.26], *P* = 0.002) with no heterogeneity (*I*^2^ = 0%, df = 3, χ^2^ = 1.94, *P* = 0.58).

### Improvement of total scores of dyspeptic symptoms after acupuncture

**Figure 4:**
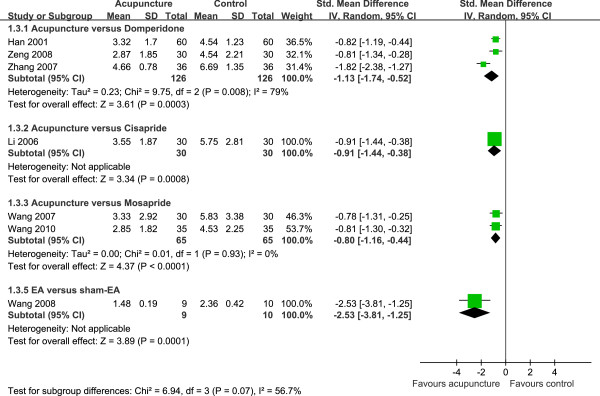
Effect of acupuncture on improvement of dyspeptic symptoms compared with control group CI, confidence interval; df, degree of freedom; M-H, Mantel-Haenszel test.

There were eight trials [21,22,24,26-29,32] observing the effect of acupuncture to improve dyspeptic symptoms in 521 patients. Acupuncture was applied for 4 [24] to 60 [29] sessions (Table 1). One study [32] with 60 cases was excluded from further analysis because this study used a different scale to score epigastric fullness syndromes which resulted in a significant heterogeneity among these eight studies. Three trials [21,28,29] evaluated acupuncture effect against domperidone in 252 patients. The random model showed that the pooled effect was statistically significant (SMD, –1.13 [95% CI, –1.74 to -0.52], *P* = 0.0003) (Figure 4). One trial [22] including 60 patients investigated the effect of acupuncture controlled by cisapride and the acupuncture effect was significant (SMD, –0.91 [95% CI, –1.44 to -0.38], *P* = 0.0008). Two trials [26,27] reported the efficacy of acupuncture in 130 patients compared with mosapride and the pooled estimate was also significant (SMD, –0.80, [95% CI, –1.16 to -0.44], *P* < 0.0001) with no heterogeneity (*I*^2^ = 0%, df = 1, χ^2^ = 0.01, *P* = 0.93). One study [24] compared the efficacy of EA with that of sham-EA, which involved 19 patients, however, it is the only trial with relative low risk of bias (Figure 2). There was a significant difference (SMD, –2.53 [95% CI, –3.81 to -1.25], *P* = 0.0001) between EA and sham-EA group (Figure 4).

### Effect of acupuncture on single dyspeptic symptom

**Figure 5:**
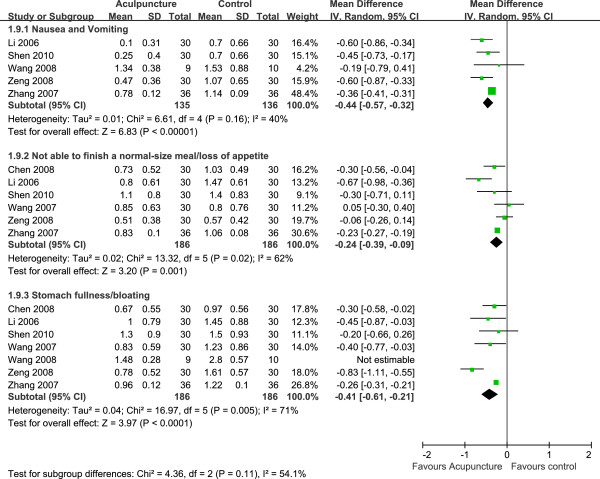
Forest plot of acupuncture effect on single dyspeptic symptom compared with control group.

Seven trials [19,22-24,26,28,29] compared the effect of acupuncture on single dyspeptic symptom with that of control groups. We classified dyspeptic symptoms into three classes, nausea and vomiting, not able to finish a normal-size meal/loss of appetite and stomach fullness/bloating according to the semiology of Chinese medicine. Five trials [22-24,28,29] involved nausea and vomiting. The pooled effect showed that acupuncture reduced the scores of nausea and vomiting when compared with control group (MD, –0.44 [95% CI, –.057 to -0.32], *P* < 0.00001, random model) with no significant heterogeneity (*I*^2^ = 40%, df = 4, χ^2^ = 6.61, *P* = 0.16) (Figure 5). Six studies [19,22,23,26,28,29] recruiting 372 patients observed acupuncture effect on improving symptoms of not able to finish a normal-size meal/loss of appetite. The pooled effect of acupuncture on these symptoms were significant (MD, –0.24 [95% CI, –0.39 to -0.09], *P* = 0.001, random model) indicating that acupuncture improved loss of appetite and help to restore patient’s food-intake. Seven trials [19,22-24,26,28,29] including 372 patients compared the effect of acupuncture on stomach fullness/bloating with that of control groups. One trial used GCSI scale showed that EA was more effective than sham-EA (MD, –1.32 [95% CI, –1.72 to -0.92]). The combined result from remaining trials was encouraging with significant difference (MD, –0.41 [95% CI, –0.61 to -0.21], *P* < 0.0001, random model) (Figure 5).

### Effect of acupuncture on gastric emptying

**Figure 6:**
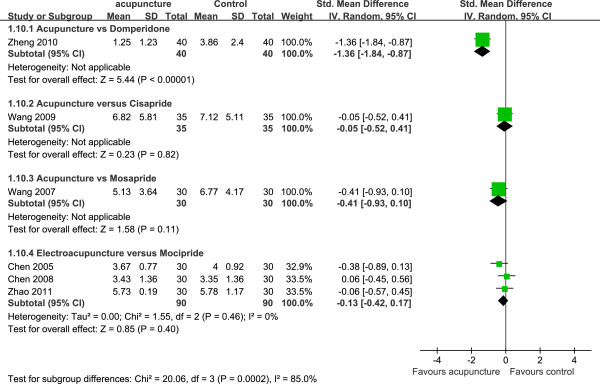
**Effect of acupuncture on solid gastric emptying with detained pellets in stomach.** CI, confidence interval; IV, inverse variance.

Seven trials [19,24-26,30-32] observed acupuncture effect on gastric emptying. One trial [24] measured gastric emptying by scintigraphy. EA significantly improved gastric emptying (SMD, –45 [95% CI, –86.02 to -3.98, *P* = 0.03, n = 9), while Sham-EA had no effect on patient’s gastric emptying. Six trials [19,25,26,30-32] recruiting 390 patients measured solid gastric emptying by radio-opaque markers. There was significant heterogeneity across six trials (SMD, *I*^2^ = 77%, df = 5, χ^2^ = 21.62, *P* = 0.0006). Three trials [19,31,32] investigated the effect of EA in 180 patients and the pooled result showed that EA had no beneficial effect (SMD, –0.13 [95% CI, –0.42 to 0.17], *P* = 0.46) compared with mosapride (Figure 6). There was no significant difference among acupuncture and cisapride (SMD, –0.05 [95% CI, –0.52 to 0.41, *P* = 0.82; one trial [25], n = 70), and mosapride (SMD -0.41 [95% CI, –0.93 to 0.10, *P* = 0.11; one trial [26], n = 60) associated with solid gastric emptying. However, acupuncture seemed to be more favorable than domperidone in patients because acupuncture improved solid gastric emptying (SMD, –1.36 [95% CI, –1.84 to -0.87], *P* < 0.00001; one trial [30], n = 80) (Figure 6).

### Adverse events

Four out of the 14 trials attempted to observed adverse events [22,24,26,29]. No adverse effect was found in acupuncture group, while in cisapride and mosapride group, some mild side effects such as dry mouth, dizziness, diarrhea, debilitation, erythra were observed [22,26].

### GRADE evidence profile

Details of GRADE evidence profile and summary of finding table were given in Table 2 and Additional file 1: Table S1, respectively. Because of serious risk of bias in study methods, heterogeneity and reporting bias, three overall qualities of evidence for response rate, total scores of gastroparesis symptoms and solid gastric emptying were judged as low quality and very low quality evidence, indicating that these estimates were uncertain and further studies are likely to have an impact on our confidence in the estimate of acupuncture effect.

**Table 2:** GRADE evidence profile for assessment of evidence quality in GRADE system

**Quality assessment**							**No of patients**		**Effect**		**Quality**	**Importance**
**No of studies**	**Design**	**Risk of bias**	**Inconsistency**	**Indirectness**	**Imprecision**	**Other considerations**	**Acupuncture**	**Control**	**Relative (95% CI)**	**Absolute**		
**Response rate to acupuncture**												
8	Randomised trial	Serious	No serious inconsistency	No serious indirectness	No serious imprecision	Reporting bias	272/292 (93.2%)	227/293 (77.5%)	RR 1.2 (1.12 to 1.29)	155 more per 1000 (from 93 more to 225 more)	⊕⊕ΟΟ LOW	CRITICAL
**Improvement of total scores of gastroparesis symptoms by acupuncture**												
6^a^	Randomised trial	Serious	No serious inconsistency^b^	No serious indirectness	No serious imprecision	Reporting bias	221	221	-	SMD 0.97 lower (1.27 to 0.68 lower)	⊕⊕ΟΟ LOW	CRITICAL
**Improvement of solid gastric emptying**												
6^c^	Randomised trial	Serious	Serious^d^	No serious indirectness	No serious imprecision	Reporting bias	195	195	-	SMD 0.37 lower (0.79 lower to 0.05 higher)	⊕ΟΟΟ Very LOW	CRITICAL

Key: ^a^, two trials [24,32] were excluded because one study [32] used a different scale to evaluate epigastric fullness syndromes and another study [24] used sham-acupuncture as control which was considered not be suitable to combine with other results. ^b^, no significant heterogeneity across these six studies was observed (SMD, *I*^2^ = 55%, df = 5, χ^2^ = 10.99, *P* = 0.05). ^c^, six studies [19,25,26,30-32] were included and one trial [24] was excluded because this study used scintigraphy to determine gastric emptying which was different from the others. ^d^, there were significant heterogeneity (SMD, *I*^2^ = 77%, df = 5, χ^2^ = 21.62, *P* = 0.0006) among these six trials.

## Discussion and conclusions

To the best of our knowledge, this is a comprehensive systematic review and meta-analysis of the effectiveness of acupuncture for diabetic gastroparesis. Our results suggested that acupuncture had a higher response rate, was more effective on improving dyspeptic symptoms including nausea/vomiting, not able to finish a normal-size meal/loss of appetite and stomach fullness/bloating, but had no significant effect on improving gastric emptying when compared with control groups. For response rate to acupuncture, some studies were in unclear risk of bias of selection bias, we conducted sensitivity analysis to trials with low risk bias of random sequence generation and the results demonstrated that response rate of acupuncture was still higher than that of control groups.

These results were encouraging, but not convincing because most trials were in unclear or high risk of bias, which is likely to overestimate treatment effect. These bias included selection bias, detection bias, attrition bias. In addition, publication bias could be a contributor of positive results since Chinese journals tended to publish RCTs with positive results [33]. No multicenter, large-scale, high quality RCTs were found. Even favorable effects on response rate to acupuncture, total scores of dyspeptic symptoms and single dyspeptic symptom were observed, we should keep in mind that these outcomes might be subjective because expectancy of patients who were willing to adopt acupuncture might overestimate acupuncture effects, especially there was no adequate control of sham-acupuncture. So the significant effects on response rate, improvement of dyspeptic symptoms might be associated, at least partly, with the less rigorous methodology of trials. Therefore a definite conclusion about whether acupuncture is effective for diabetic gastroparesis cannot be drawn from current trials because of the low quality of included trials.

According to GRADE system, the evidence of acupuncture for diabetic gastroparesis was assessed as low quality and very low quality. Therefore, the routine use of acupuncture in the treatment of diabetic patients with gastroparesis was not recommended. This result was in line with a updated clinical guideline for management of gastroparesis [34] in which acupuncture was assessed as low level evidence and has been recommended for conditional used for gastroparesis as an alternative therapy.

Because majority of the trials reported very limited details of study design and performance, we attempted to contact authors by letters or e-mails for additional information, but we got a few responses. Reporting methodology of all trials except one [24] were inconsistent with the extending CONSORT statement about the standards for reporting interventions in clinical trials of acupuncture (STRICTA) [35], although this statement has been stressed in many journals in Chinese and English. So it is still necessary to emphasize these statements to editors of journals, acupuncturists and medical students.

In China, it is a big challenge to blind participants and personnel in acupuncture practice because many patients have an experience of acupuncture or know that Deqi is necessary in acupuncture, which makes the sham-acupuncture impossible in practice. But blinding outcome assessors and statisticians is feasible and should be adopted in future studies.

As previous reported [36,37], many Chinese trials used to classify acupuncture efficacy to three or four grades, clinical cure, significant improvement, improvement and no improvement. Because this outcome measure is not easy to understand for international colleagues and may hamper the academic communication of acupuncture, outcome evaluation like this should not be adopted in future trials. GCSI was a new developed and reliable instrument to evaluate dyspeptic symptom severity in gastroparesis patients [11]. GSCI total scores were sensitive to the changes of overall gastroparesis symptoms assessed by clinicians or patients [38], so we strongly recommend using this scale to evaluate the acupuncture effect on symptom severity in the future studies.

Diabetic gastroparesis often leads poor life quality [39], assessment of quality of life should be regarded as one of outcomes using some scales such as Medical Outcomes Study (MOS) 36-Item Short Form Health Survey (SF-36), the functional digestive diseases quality of life questionnaire (FDDQL) [40] and Upper Gastrointestinal Disorders-Quality of Life [41] in the future studies.

In addition, although there is no strong evidence available to determine how many times acupuncture should be performed in one day or one week, it seems to be reasonable to conduct one session of acupuncture per day if patient’s time conditions permit because the therapeutic effect of acupuncture every session was thought to last for 4–6 hours [42,43].

In short, a definite conclusion on efficacy of acupuncture for GDP can not be drawn from this review because of the low methodological quality of the included trials. But acupuncture seems to be beneficial and real safe for diabetic gastroparesis as suggested by this systematic review. It is necessary to performed well-designed, larger-scale, placebo-controlled, long term follow-up trials to evaluate the efficacy of acupuncture for diabetic gastroparesis.

## Competing interests

The authors declare that they have no competing interests.

## Authors’ contributions

MXY conceived and designed experiment, searched databases, extracted and assessed studies, analyzed data and drafted the manuscript. XML, searched databases, extracted and assessed studies, analyzed data and drafted the manuscript. SYY and XJL conceived and designed experiment. ZPL, MX and DHG helped to search databases and analyzed data. SHL participated in discussion of results and draft the manuscript. All authors read and approved the final manuscript.

## Pre-publication history

The pre-publication history for this paper can be accessed here:

http://www.biomedcentral.com/1472-6882/13/311/prepub

## Supplementary Material

Additional file 1: Table S1 Summary of finding table produced by GRADEprofiler. Click here for file
