# Supplementary material for: Meta-analysis of acupuncture for relieving non-organic dyspeptic symptoms suggestive of diabetic gastroparesis
Source: BMC Complement Altern Med. 2013 Nov 9;13:311. doi: 10.1186/1472-6882-13-311 (PMC4226258; doi:10.1186/1472-6882-13-311)
Supplement: Additional file 1: Table S1 — Summary of finding table produced by GRADEprofiler. [file 1472-6882-13-311-S1.doc]

**Supplementary Table**

Supplementary table 1. Summary of finding table produced by GRADEprofiler.

| **Acupuncture effects for diabetic gastroparesis Controlled by** **gastroprokinetic agents** | | | | | | |
| --- | --- | --- | --- | --- | --- | --- |
| **Patient or population:** patients with diabetic gastroparesis **Settings:**  **Intervention:** Acupuncture versus Control | | | | | | |
| **Outcomes** | **Illustrative comparative risks* (95% CI)** | | **Relative effect (95% CI)** | **No of Participants (studies)** | **Quality of the evidence (GRADE)** | **Comments** |
|  | Assumed risk | Corresponding risk |  |  |  |  |
|  | **Control** | **Acupuncture versus Control** |  |  |  |  |
| **Response rate to acupuncture** (effective vs ineffective) | **Study population** | | **RR 1.2**  (1.12 to 1.29) | 585 (8 studies) | ⊕⊕⊝⊝ **low** | Acupuncture has a higher response than control group. Sensitivity analysis limited to low risk of bias in random sequence generation also showed that a similar effect (RR =1.15, [95% CI, 1.05 to 1.26], P =0.002) |
|  | **775 per 1000** | **930 per 1000** (868 to 999) |  |  |  |  |
|  | **Moderate** | |  |  |  |  |
|  | **758 per 1000** | **910 per 1000** (849 to 978) |  |  |  |  |
| **Scores of gastroparesis symptoms** |  | The mean scores of gastroparesis symptoms in the intervention groups was **0.97 standard deviations lower** (1.27 to 0.68 lower) |  | 442 (6 studies) | ⊕⊕⊝⊝  **low** | SMD -0.97 (-1.27 to -0.68) |
| **Solid gastric emptying time** |  | The mean gastric emptying time in the intervention groups was **0.37 standard deviations lower** (0.79 lower to 0.05 higher) |  | 390 (6 studies) | ⊕⊝⊝⊝ **very low** | SMD -0.37 (-0.79 to 0.05) |
| *The basis for the **assumed risk** (e.g. the median control group risk across studies) is provided in footnotes. The **corresponding risk** (and its 95% confidence interval) is based on the assumed risk in the comparison group and the **relative effect** of the intervention (and its 95% CI).  **CI:** Confidence interval; **RR:** Risk ratio; | | | | | | |
| GRADE Working Group grades of evidence **High quality:** Further research is very unlikely to change our confidence in the estimate of effect.  **Moderate quality:** Further research is likely to have an important impact on our confidence in the estimate of effect and may change the estimate. **Low quality:** Further research is very likely to have an important impact on our confidence in the estimate of effect and is likely to change the estimate. **Very low quality:** We are very uncertain about the estimate. | | | | | | |
|  | | | | | | |
